# The Dimensionality, Consistency, and Structural Validity of an Instrument Used to Measure Obesogenic Attitudes in Parents from Southern Spain (The PRELSA Scale)

**DOI:** 10.3390/nu16081135

**Published:** 2024-04-11

**Authors:** Jesús Carretero-Bravo, Mercedes Díaz-Rodríguez, Bernardo Carlos Ferriz-Mas, Celia Pérez-Muñoz, Juan Luis González-Caballero

**Affiliations:** 1 Department of General Economy, Health Sociology Area, University of Cadiz, Avda. Ana de Viya 52, 11009 Cádiz, Spain; 2 Department of Nursing and Physiotherapy, University of Cadiz, Avda. Ana de Viya 52, 11009 Cádiz, Spain; mercedes.diaz@uca.es; 3 Clinic Management Unit (UGC), Andalusian Health Service, 11510 Puerto Real, Spain; 4 Department of Mechanical Engineering and Industrial Design, University of Cadiz, Polígono Río San Pedro, 11510 Puerto Real, Spain; 5 Department of Statistics and Operations Research, University of Cadiz, Polígono Río San Pedro, 11510 Puerto Real, Spain

**Keywords:** childhood obesity, scales, parental attitudes, feeding practices, reliability and validity

## Abstract

(1) Background: We aimed to analyze the dimensionality, internal consistency, and structural validity of the Preschool Eating, Lifestyle, and Sleeping Attitudes Scale (PRELSA Scale), which is an instrument that was designed to measure obesogenic behaviors. (2) Methods: We carried out an observational study by means of an online survey. The PRELSA Scale consists of 13 dimensions and 60 items relating to the most common obesogenic behaviors and attitudes. Additionally, we obtained sociodemographic characteristics and concrete habits from the sample. We obtained the responses of 791 parents and caregivers of preschool children between 2 and 6 years of age in Andalusia (southern Spain). We analyzed dimensionality through an Exploratory Factor Analysis (EFA), consistency through Cronbach’s Alpha, structural validity through a Confirmatory Factor Analysis (CFA), and measurement invariance with multigroup CFA models. (3) Results: The EFA showed a 14-dimensional structure with 48 items. The internal consistency was acceptable in all dimensions (Cronbach’s Alpha range of 0.72 to 0.97). The structure was confirmed in the CFA with good fit indices (CFI and TLI > 0.9 and RMSEA < 0.05). We ensured that the scale had measurement invariance regarding education, income, and marital status. (4) Conclusions: The PRELSA Scale shows promising properties that have the potential to measure obesogenic behaviors in Spain, which could be the basis for future interventions associated with the prevention of childhood obesity in healthcare and educational settings.

## 1. Introduction

It is estimated that in 2030, 18.6 million children between 5 and 9 years of age will have obesity worldwide [1], leading to psychiatric and psychosocial disorders during childhood and an increased risk of developing chronic conditions as well as being adults with obesity [2,3]. In Europe, specifically in Spain and the Mediterranean countries, the situation is not better [4]. The ALADINO 2019 study showed that 40.5% of schoolchildren between six and nine years of age had overweight or obesity [5]. Interventions have been made at the national and Andalusian levels to prevent obesity, but in the 2011–2019 period, the prevalence of obesity remained stable [6,7]. Most prevention trials have focused on schoolchildren or adolescents and on adequate child growth when excess weight is often established, with unspecific recommendations that do not attempt to influence and modify parental attitudes.

If we focus on the onset of childhood obesity, one of the critical periods is the stage up to adipose rebound, which is a naturally occurring increase in BMI that usually happens at six years of age [8]. Prior to this period, earlier interventions can be developed in two periods. The first is the 1000 days period (pregnancy and the first two years), which is when the early programming mechanisms present their maximum plasticity, and interventions in this period have shown promising results for childhood obesity prevention [9,10]. The second period is later, in the preschool age, which is when acquiring and maintaining healthy habits and lifestyles are significant to avoid an early adipose rebound, which has a higher risk of future obesity [8].

During this preschool period, children experience rapid physical and cognitive growth, and their bodies are extremely sensitive to external influences. Dietary patterns, physical activity levels, and sedentary behaviors could significantly impact a child’s future health [11,12]. Most of the risk factors in this period are considered modifiable or educable [13], where the role of parents and caregivers is crucial in shaping the development of children. The thoughts, beliefs, attitudes, and habits of parents significantly impact children’s growth, particularly concerning childhood obesity [11,14]. Parents directly influence the alleviation of overweight in children by promoting appropriate values and serving as role models [15,16]. Furthermore, a meta-analysis indicated that interventions targeting parental attitudes during the preschool stage yield positive outcomes in terms of BMI [17]. 

Taking this into account, a good starting point to act on childhood obesity and develop future targeted interventions can be to measure parental attitudes that may lead to this condition. The habits mainly associated with the prevention of obesity are an adequate diet, physical activity, avoiding sedentary behavior, controlling screen viewing, and establishing good sleep routines [18,19,20,21]. There are several instruments that measure the attitudes associated with these habits in parents and caregivers of children [22,23,24,25], although they are not so common in the preschool stage.

In addition, the instruments that have attempted to measure these attitudes in Spain have been characterized by being based on specific habits, and not so much on parents’ and caregivers’ attitudes or beliefs [26,27,28]. Some instruments only focus on feeding or physical activity, and do not have a broad perspective that also considers all the obesogenic attitudes [29,30,31,32], especially at the preschool age. Given this need, our research team conducted a pilot study to construct an instrument that could cover all obesogenic behaviors aimed specifically at parents and caregivers with preschool children: the Preschool Eating, Lifestyle and Sleeping Attitudes Scale (PRELSA Scale) [33]. This instrument was developed based on the opinions of experts in the field and by drawing on several of the most frequently used instruments, as discussed extensively in our first paper.

The development of this instrument was carried out with a double initial objective: obtaining two scales, including a complete one, which attempts to measure the dimensions as accurately as possible, and a shorter one, which contains the key items and is easy to use in settings where there is limited time available, such as educational institutions or primary care settings. Thanks to this pilot test, it was possible to ensure the scales’ interpretability, feasibility, and content validity while obtaining an instrument with 60 items and 13 dimensions, which responds to parental attitudes associated with obesogenic behaviors. Under this initial conceptual framework of the scale, this article shows the first results of the field test of the PRELSA Scale on a sample of parents and caregivers in Andalusia (South Spain) with the aim of analyzing some of its psychometric properties (dimensionality, internal consistency, and structural validity). These first properties will be the basis for achieving a valid and reliable extensive instrument with which to subsequently determine the accuracy of a shorter instrument.

## 2. Materials and Methods

### 2.1. The PRELSA Scale

This study shows the results of the field test of the PRELSA Scale. This instrument was previously validated in a pilot test, in which its content validity and initial conceptual framework could be assured [33]. In this first study, a research team with a pediatrician (B.C.F-M.), a physician (M.D-R.), a doctor in statistics (J.L.G-C.), a researcher in statistics (J.C-B.), and a researcher in health sciences (C.P-M.) created an initial version of the instrument by analyzing existing scales and incorporating items and dimensions that were considered necessary. The instrument obtained in this pilot test consisted of 60 items divided into 13 dimensions. 

**Figure 1:**
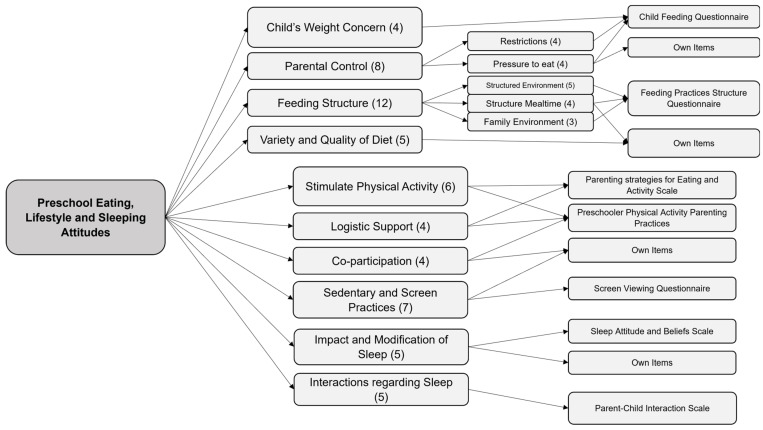
Initial conceptual framework of the PRELSA Scale. The numbers in parentheses indicate the number of items in each dimension.

The items in these dimensions came from already validated scales regarding feeding practices (Child Feeding Questionnaire and Feeding Practices and Structure Questionnaire [34,35]), physical activity and sedentary practices (Parenting Strategies for Eating and Activity Scale (PEAS) and Preschool Physical Activity Parenting Practices Scale [36,37]), screen viewing [38] and sleep routines (Sleep Attitudes and Beliefs Scale (SABS) and Parent–Child Sleep Interactions Scale (PSIS) [39,40]), as well as other self-developed items. The aim was to cover the dimensions that the team considered fundamental regarding obesogenic attitudes, expanding on some items that our team considered important in the current context and the situation of parental attitudes in our country. Figure 1 shows the conceptual framework developed after the pilot test and the instruments from which the items originated.

The original scales were used as the basis of the items while respecting the original meanings. However, the way of expressing them was transformed so that they were all associated with parental beliefs and attitudes and not with concrete actions. The original version of the scale in Spanish with 60 items and the translation for this paper in English can be found in the Supplementary Material. The response categories in the scale items are in Likert format with values ranging from one to five, with one being disagree and five being agree, except in the Child’s Weight Concern dimension, where one indicates not at all concerned and five indicates very concerned. Most items are formulated so that a score of five is the appropriate response in terms of attitude or habit. However, some items are formulated in reverse to avoid repetitive response bias. 

The complete questionnaire used in this field test consisted of two other sections. The first section consisted of 24 questions in which the parents or caregivers responded to physical characteristics (sex, age, weight, height, or number of family members), socio-economic status (family income, level of studies, work situation, or marital status), and characteristics of their environment (square meters of the home, outdoor spaces, or location of the home). The last section comprised 26 questions, where we asked about the children’s specific behaviors and habits, such as the intake of sugary foods, duration of physical activity, screen-viewing hours, or hours of night-time sleep. These questions were formulated by considering the fundamental guidelines of the World Health Organization (WHO) to prevent childhood obesity [41]. 

### 2.2. Participants and Data Collection

Data collection was conducted online from October 2022 to May 2023. Originally, the data collection step was going to be carried out face-to-face in primary care centers, but the overload of health care centers in Andalusia made it necessary to change the sampling method. The survey was only distributed and disseminated through online media, and the recipients were parents and caregivers who had a child in their care aged between 2 and 6 years old (preschool age in Spain) and were residents of Andalusia. These digital media were parents’ forums and associations, websites of schools and nurseries, educational institutions, and official media associated with children. Educational institutions and official media were invited to disseminate the survey among all potential recipients using the social media accounts or internal emails of each institution.

The questionnaire was designed on the online survey platform LimeSurvey (5.2 version) [42] and was self-administered by each user. At the beginning of the questionnaire, the users were explicitly asked to complete the questionnaire only if they fulfilled the selection criteria. If not, the end of the questionnaire was displayed on the screen. In addition, we requested acceptance of their participation and the collection of anonymized survey data. The content and information shown to the participants were written according to the pilot test results so that the users clearly knew whether they were recipients or not. The LimeSurvey platform was also configured so that only one response per participant IP could be obtained. 

A total of 1799 people started the online survey during the collection period. Of these people, 906 completed the survey. Those who indicated a postcode not belonging to Andalusia were discarded, so the final sample size was 791 individuals. The average time taken to complete the survey was 18.56 min. 

### 2.3. Data Analyses

We followed the procedure suggested by the Consensus-based Standards for the Selection of Health Measurement Instruments (COSMIN) [43].

First, we reviewed the sociodemographic characteristics of the sample through the most common statistics (means and deviations in continuous variables, percentages, and frequencies in discrete variables). We first analyzed the distribution of the response categories in the 60 items, determining the frequencies, missing values, mean, variance, and skewness of each item. We also observed the response pattern, the deviation between responses, the mean, the number of missing responses, and the time taken to answer the questionnaire to detect possible individuals with anomalous responses.

We also checked the correlation matrix of the items using the polychoric correlation coefficient, which is suitable for Likert scale variables [44], to determine which items could be discarded from the scale prior to the dimensionality analysis based on four criteria: too many missing items (more than 10%), high item correlations (greater than 0.85), response categories with high frequencies (more than 90%), and high skewness values (more than 3) [45]. Using the initial items, a dimensionality analysis was carried out through an Exploratory Factor Analysis (EFA) with the Lavaan package in R. Beforehand, we calculated the KMO statistic and Bartlett’s test of sphericity to determine the adequacy of the reduction in dimensions, looking for a KMO of at least 0.8 and a significant *p*-value in the test. After this, we checked the number of factors in the EFA, considering the criterion of eigenvalues greater than one and the fact that the original conceptual framework had 13 dimensions, to determine the number of dimensions. We used the WLSMV (Weighted Least Squares Mean and Variance) estimator as a factor extraction method [46].

The models obtained with 12 to 14 dimensions and different numbers of items were analyzed in search of the one with the best-fit indices and the best conceptual meaning in our context. We also tested the best solution with various oblique rotations (Oblimin, Geomin, Bentler, and Quartimin). The fit indices used were the Comparative Fit Index (CFI), the Tucker–Lewis Index (TLI), and the Root Mean Square Error of Approximation (RMSEA) in their scaled versions. Items with factor loadings of less than 0.3 and communalities of less than 0.2 were deleted, and items with factor loadings between 0.3 and 0.35, communalities between 0.2 and 0.3, or possible cross-factor loadings were reviewed.

With the obtained model in the EFA, we analyzed the reliability from its internal consistency. We reviewed Cronbach’s Alpha coefficient and sought to find coefficients ranging from 0.7 to 0.95 to determine a good fit [47,48]. These coefficients were calculated on the polychoric correlation matrix following the recommendation for Likert scale items with five response categories [49]. 

Finally, we checked the structural validity of the obtained dimensional model through a Confirmatory Factor Analysis (CFA). We used polychoric correlations and the WLSMV estimator as a factor extraction method. The usual CFA criteria (CFI > 0.9 and TLI > 0.9, but preferably > 0.95 and RMSEA < 0.5) were taken as indicators of good fit, and we used the scaled versions of the indices due to the extraction method used. Once the CFA model was obtained, we determined items with low factor loadings (below 0.4) and low communalities (below 0.2) as candidates to be eliminated. We also reviewed the modification indices provided by Lavaan to analyze possible items in other dimensions or possible correlations to be added to the model.

In addition, the measurement invariance of the CFA model was calculated for three variables of interest, namely education, income, and marital status, and discarded sex due to the low sample size for men. Multigroup CFA models were successively estimated by adding restrictions to certify the invariance. The first model, the configural model, assumed only the multigroup information; the second, the metric model, assumed equality between the factor loadings of each group; and the third, the scalar model, assumed equality between the factor loadings and intercepts. The three models were compared by checking the difference between the CFI and RMSEA indices; CFI values ≤ |−0.005| and changes in RMSEA ≤ 0.010 when we had an unequal sample size within each group implied measurement invariance [50,51].

All of the analyses were conducted using R (version 4.1.2, the R Foundation for Statistical Computing). To perform the EFA and CFA, we used the Lavaan package version 0.6-16 [52], which incorporates the most up-to-date estimators for categorical variables and allows us to obtain both standard and scaled CFA fit indices.

## 3. Results

The final sample consisted of 791 parents and caregivers. Table S1 shows their main characteristics. It highlights that there was a high number of women (93.3%) and higher educational and purchasing levels compared to the average in this region (more than 50% with an income over EUR 2000 and more than 60% with university studies). The mean age of the respondents was 37.95 years, and it should also be noted that, on average, the respondents were overweight (BMI = 25.53 > 25).

### 3.1. Dimensionality

We first conducted a descriptive analysis of the items (Table 1) and correlations. This descriptive analysis shows 20 candidate items for deletion, from which we decided to eliminate CWC1 (a category with more than 90%), RES1 (it had a correlation with RES2 higher than 0.9, so we chose RES2 due to it having slightly more variability in the responses), LSA1 (a category with more than 90%), and IMS2 and IMS3 (they had missing values above 20%), so the EFA started with 55 items. The analysis of the response pattern, response time, and mean and variance of the responses did not detect any individual with anomalous responses. However, two individuals were found to have more than 15 unanswered items, so their responses were eliminated, leaving a final sample size of 789.

Prior to the EFA, we also analyzed the adequacy of the data for such an analysis through Bartlett’s test of sphericity (*p*-value < 0.001) and the Kaiser–Meyer–Olkin statistic (KMO = 0.83), with results that showed adequacy for the dimension reduction analysis. Table 2 shows the successive factor analyses performed. Initially, solutions of 12 to 14 dimensions were tested. We decided to choose the 14-factor solution, since it made structural sense once the rotation had been carried out and presented good fit values. Regarding the rotation, the Bentler rotation was found to have the most meaningful structure and the least cross-factor loadings.

In this first model with 14 dimensions and 55 items, 3 items were found with factor loadings of less than 0.3, which were eliminated (IRS2, IRS4, and SBI1). We also deleted one item (CWC3) with a communality bigger than 1 for convergence problems. In the new model with 51 items, 1 item was found with cross-factor loadings that were difficult to interpret (IRS5), three items with factor loadings lower than 0.35 (VQD5, SV2, and SMT3), and one item with a communality lower than 0.25 (IRS3). After a review by the team, IRS5, VQD5, and SV2 were deleted, keeping SMT3 and IRS3 because they belong to dimensions with few items. Finally, the model with 48 items and 14 dimensions (Table S2) was chosen, and the RMSEA, CFI, and TLI indices were consistent with an optimal fit, with a percentage of variance explained of 61.3%. The 14 dimensions ranged in size from two to eight items.

With respect to the initial conceptual framework, we found some relevant changes in the structure. We found a new dimension, and several of the items changed compared to the original factor. In addition, some of the dimensions were found to have changed meanings with respect to the original framework. The changes will be reviewed in depth in the Discussion Section.

### 3.2. Internal Consistency

Cronbach’s Alpha reliability coefficients were calculated on the polychoric correlation matrix of the 48 items. To determine the scores, the order of the items was changed so that all items reflected that a score of five on the Likert scale reflects the correct value for each item; therefore, parents with high scores for each dimension could be identified as parents with appropriate obesity avoidance behaviors. The results can be seen in Table 3.

Looking at the factors, it is important to highlight the F1, F3, and F6 dimensions, which have mean item values below four, showing that these are dimensions in which parents show attitudes and beliefs that are further away from those that should be adequate, unlike the other dimensions where the average is close to five. Regarding internal consistency, all dimensions show adequate reliability coefficients (above 0.7), with the F14 dimension showing a lower but acceptable value of 0.72. It should be noted that eight dimensions have a coefficient higher than 0.95, namely F5, F9, and F10. The total scale has a Cronbach’s Alpha of 0.89. 

### 3.3. Structural Validity

Once the factorial model was established, we analyzed the structural validity of the new model. For this purpose, the model derived by the previous EFA (48 items and 14 dimensions) was tested using a Confirmatory Factor Analysis (CFA) under the pretext of a simple structure, i.e., that each item belonged to only one dimension. If we look at Table S2, we can see that item SV4 has cross-factor loadings above 0.35 on two dimensions and item SPA3 has factor loadings above 0.3 on three dimensions. For consistency with the dimensions, we decided to include item SV4 in dimension F2, which is associated with the use of rewards, and item SPA3 in dimension F8, which is associated with the importance of physical activity. The results can be seen in Table 4.

**Figure 2:**
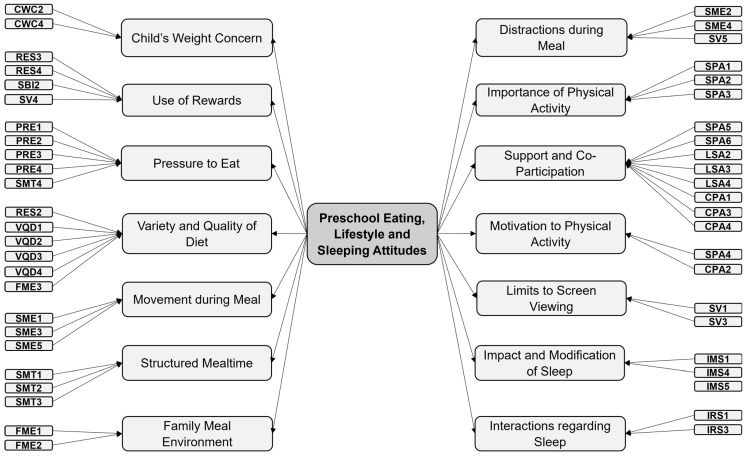
Factorial structure of PRELSA Scale after CFA.

The confirmatory model yielded acceptable results. The FME4 item had a communality of less than 0.2, but we decided to keep it because it made conceptual sense and because the fit indices were good. The model with 14 factors has a CFI of 0.929 and a TLI of 0.918 (bigger than 0.9), while it has an RMSEA of 0.034 with a confidence interval of (0.031, 0.036), which also indicates a good fit. In Figure 2, we can see the final name proposed for each dimension and the items associated with each factor. The complete scale with the 48 items and 14 dimensions can be found in the Supplementary Material.

Under this structural model, we are interested in analyzing measurement invariance regarding variables that have an influence on parents and caregivers. We carried out an invariance analysis on educational level, income level, and marital status, and the results are shown in Table 5. For educational level, we compared university and non-university students; for income level, we compared individuals with incomes of less than EUR 2000 and those with incomes of more than EUR 2000; and for marital status, we compared married and unmarried individuals.

In all three models, we can ensure configural invariance, as all three configural models have acceptable fit indices. Furthermore, we can ensure both metric and scalar invariance, as the differences in the CFI and RMSEA are in the acceptable range within the usual criteria.

## 4. Discussion

In this study, we developed the steps taken in the field test to analyze the first psychometric properties of the PRELSA Scale. The importance of this study lies in the fact that it is one of the first in Spain, specifically in Andalusia, to validate a psychometric instrument in the fundamental period of children who are 2 to 6 years (preschool age) by measuring the beliefs and parental attitudes that could lead to future obesity. Due to the length of the scale, this first analysis of the properties reviews their consistency, dimensionality, and structural validity, which will serve as a basis for future studies to test their definitive construct validity, as well as their interpretability and relationship with other measures of obesity.

The final analysis sample for the field test consisted of 791 participants. Regarding the sample, the vast majority, more than 90%, was female. Also, 99% of the respondents were the parents of the children to whom the survey referred, and in general, they had higher socio-economic levels than the average in Andalusia, especially in terms of educational attainment and income level. These data show two key aspects. The first is associated with the high percentage of women, as the survey was freely disseminated in parent groups, childcare forums, or emails from schools and educational institutions. The fact that most respondents were women shows that women carry the burden of childcare and are the ones who show interest and attach importance to responding and participating in studies of this type [53].

On the other hand, we reached parents with higher socio-economic statuses than the average. We must consider the bias of access through online media, which sometimes makes access difficult for the most disadvantaged sectors of society [54]. Another reason could be that parents with higher levels of education or income may be more interested in childcare and in responding to this type of survey. Even so, 17.8% of parents with basic education levels and 7.3% with incomes below EUR one thousand participated in this study, although face-to-face sampling in cross-cutting socio-economic areas such as health centers and Andalusian public schools could solve this problem. It is also important to mention that the average BMI was above 25, showing that parents struggle with the problem of being overweight, which is a factor that predisposes children to possible obesity [55].

In the initial pilot test of the scale, its content validity was analyzed, and a conceptual framework was established. Through the analysis of dimensionality and structural validity, this study allowed us to certify the conceptual framework that is latent behind the items and dimensions of the PRELSA Scale. Concerning the initial conceptual framework involving 60 items and 13 dimensions, the exploratory factor analysis ended up showing a structure of 48 items and 14 dimensions. The modifications made to the scale framework seem to make acceptable conceptual sense; even with the changes, the dimensions are conceptually similar to those seen in the original feeding scales [34,35] and cover the aspects that were considered by the team to be the most determinant.

Regarding the feeding items, the Restrictions dimension was limited to items associated with rewards, which included items that were related to factors other than feeding attitudes, such as the use of screens. The original Structured Meal Environment dimension was split into two sub-dimensions, one associated with movement during the meal and the other associated with pure distractions. This change may stem from the fact that certain attitudes have been separated that differentiate parents with more authoritative styles when it comes to monitoring feeding time. In addition, one item from the Family Meal Environment dimension was eventually associated with the Pressure to Eat dimension, and another was associated with the dimension regarding Variety and Quality of Diet. For these changes, we must consider that when mixing two scales, it is normal for some items to be interspersed [56], given that the Child Feeding Questionnaire did not have certain dimensions associated with the structure of the feeding time in its conception that are in the Feeding Practices and Structure Questionnaire. What is important for our scale is to maintain the conceptual meaning of the dimensions found.

When it came to physical activity and sedentary attitudes, a systematic review established that a tool that measures these attitudes should at least have dimensions associated with the ability of parents to stimulate physical activity, provide logistical support for the activity, and co-participate in the activities [22]. The dimensions set out in the initial conceptual framework were transformed such that the items that were more properly associated with co-participation and logistical support for activity were merged into one dimension, with eight items with unique factor loadings in this factor. On the other hand, two other dimensions were associated with items regarding the perceived importance of physical activity and the prominence given to it compared to other types of activities.

Two items associated with limits on screen viewing emerged in one dimension, while the other items related to screens were associated with other factors. It was therefore seen that screen habits are cross-cutting, showing how screens have become a fundamental part of various aspects of everyday family life and how appropriate ways of using them should be introduced into the dimensions associated with family behaviors.

The dimensions associated with sleep remained similar, albeit with fewer items, since two of them that were eliminated at the beginning of the analysis (IMS2 and IMS3) had a high percentage of missing items. Concerning these sleep dimensions, it is important to note the problem with missing values, which may indicate that, in this aspect of the relationship between obesity and sleep attitudes, parents’ knowledge is more deficient [57].

The internal consistency of the scale was more than acceptable, with an overall Cronbach’s Alpha of 0.89, and it was also acceptable in each dimension, ranging between 0.73 and 0.97. We should highlight three factors with a consistency of 0.97, which exceeded the range of 0.95, which may have been due to redundancies in the items [48]. After reviewing the items in these dimensions, we believed that in this first extensive version of the scale, they were not redundant, but this fact is a good indicator of possible dimensions in which to reduce items for the brief instrument that we want to develop. The scales on which our items are based had similar reliability results [36,37,39,40,58,59], although they are different instruments, with the dimensions associated with feeding having higher Alpha coefficients than those of physical activity and, above all, sleep. Moreover, as there were five dimensions with two items, it was expected that they would present somewhat lower reliability results.

Finally, the Confirmatory Factor Analysis showed that the scale structure presents fit indices close to what is desirable in this type of analysis. The sample size obtained, 791 participants, was enough compared to the usual recommendations for conducting a CFA (at least 200 participants and 10 cases per item; in our case, we had a sample size of 600 [60]). The scaled CFI and TLI remain in the range between 0.9 and 0.95, which indicates an acceptable fit to the standard criteria [61] and, as an instrument under construction, is considered a good initial indicator. In addition, the scaled RMSEA is less than 0.05, indicating optimal adequacy. In this confirmatory structure, it is important to highlight the fact that we found five dimensions with two items. Given that the dimensions should ideally have at least three items, this suggests that a second-order factor structure might be appropriate to simplify the number of dimensions, improve the interpretability of the scale, and mitigate the lack of items in the five factors with two items.

In addition, the measurement invariance for three socio-economic variables relevant to the family environment, such as education, income, and marital status, was confirmed. It was shown that these three variables influence the way parents act with respect to the problem of childhood obesity [62,63], and this invariance allows for comparisons of scores on this scale between these groups of parents to be made in subsequent analyses. Also, to ensure gender invariance, it will be necessary to reach a larger group of fathers, which, in view of the low level of interest shown in this first field test, will require specific sampling (probably face-to-face sampling).

This study has some relevant strengths. To our knowledge, it is one of the first in Spain to measure family obesogenic behaviors at preschool age from a broad perspective. Considering the field of this study, the sample size was large, and the results obtained confirm that the factor structure and consistency of the items are adequate.

However, there are some limitations that are important to note. As the sample was obtained online, it was not possible to access families with difficulties in accessing the Internet, which may be one of the reasons why we obtained a sample with a higher-than-average social level in Andalusia. Online sampling also made it challenging to analyze reliability from a test–retest perspective, which would make it possible to measure the consistency of the instrument over time. One way to solve this limitation would be to conduct future studies with the already validated scale by trying to return to the original method of face-to-face sampling in primary care centers. Finally, there are five dimensions with two items, which is not advisable in factorial structures and can be solved in future work by proposing second-order factorial structures that group similar dimensions.

## 5. Conclusions

Childhood obesity is a challenge that needs to be addressed from the earliest stages of development, which is a time when family plays a key role. Preventive interventions at the preschool age need to be developed with a thorough knowledge of all aspects associated with obesogenic behaviors in the family. To this end, it is necessary for all of the instruments involved in these types of interventions to be as accurate and reliable as possible.

This study can serve as a basis for the knowledge of parental and family attitudes in future interventions proposed by educational or health institutions in Spain. Although instruments for measuring these obesogenic attitudes already existed in other languages, no tools based on previously validated factors that also opt for a perspective focused on parental attitudes and beliefs had been developed so far in our environment, and this work could provide a tool for the institutions involved in prevention.

To this end, the PRELSA Scale has shown a promising dimensionality, structure, and consistency for measuring obesogenic behaviors. Under this premise, it is necessary to confirm these suitable properties by analyzing, in future studies, its construct validity, its interpretability, and its relationship with measures that determine obesity to certify that is adequate to measure obesogenic behaviors in preschool families. 

Furthermore, once these properties have been certified, we aim to develop a second instrument, based on the first one, which will briefly measure the most psychometrically significant obesogenic aspects. This second instrument will facilitate the application of the PRELSA Scale in settings such as primary care settings, where it is necessary to make the most of the limited time available.

## Figures and Tables

**Table 1:** Descriptive analysis of PRELSA Scale involving 60 items.

Row	Missing	1	2	3	4	5	Mean	SD	SKEW
CWC1	0.00	0.86	6.65	**90.39**	1.97	0.12	2.94	0.34	−2.34
CWC2	0.00	66.63	18.47	8.87	3.69	2.34	1.57	0.97	1.82
CWC3	0.00	39.66	16.87	16.13	11.45	15.89	2.49	1.5	0.5
CWC4	0.00	30.05	15.15	17.49	13.18	24.14	2.88	1.56	0.12
RES1	0.38	0.74	1.11	1.11	7.27	89.41	4.84	0.56	**−4.51**
RES2	0.25	0.99	1.23	1.97	8.13	87.44	4.8	0.63	**−3.99**
RES3	0.88	50.25	17.73	5.42	17.36	8.37	2.15	1.41	0.82
RES4	0.51	78.69	11.33	2.96	5.05	1.48	1.39	0.89	2.5
PRE1	0.38	47.91	18.35	4.19	21.55	7.64	2.21	1.42	0.7
PRE2	0.88	14.04	8.99	2.34	25.25	48.52	3.85	1.47	−1.01
PRE3	0.63	48.03	21.67	1.48	18.97	9.24	2.18	1.43	0.82
PRE4	1.14	27.09	10.84	1.85	25.25	33.87	3.29	1.66	−0.37
VQD1	0.38	1.48	2.34	0.99	6.90	87.93	4.79	0.71	**−3.93**
VQD2	0.38	62.32	24.88	2.34	7.88	2.09	1.62	1.01	1.76
VQD3	0.76	3.57	6.77	2.22	25.74	60.96	4.35	1.06	−1.82
VQD4	0.51	0.62	1.11	0.86	9.61	87.32	4.83	0.55	**−4.26**
VQD5	1.39	31.77	24.01	3.08	24.51	15.15	2.67	1.52	0.28
SME1	0.13	68.47	17.12	4.93	6.16	3.20	1.59	1.05	1.87
SME2	0.25	58.13	19.70	7.51	10.84	3.57	1.81	1.18	1.3
SME3	0.51	2.09	6.77	4.31	20.94	65.39	4.41	1.01	−1.82
SME4	0.13	67.61	15.02	5.30	9.61	2.34	1.63	1.08	1.65
SME5	0.63	60.34	20.32	4.56	8.25	5.91	1.77	1.21	1.52
SMT1	0.13	33.74	29.31	1.23	21.55	14.04	2.52	1.49	0.49
SMT2	0.38	16.38	27.46	5.91	33.62	16.26	3.05	1.39	−0.1
SMT3	1.01	1.97	5.05	2.34	26.97	62.68	4.45	0.92	−2.01
SMT4	0.76	68.23	17.86	8.74	3.69	0.74	1.49	0.86	1.82
FME1	0.76	1.11	1.72	4.06	13.55	78.82	4.69	0.73	−2.85
FME2	0.76	4.80	2.71	2.46	16.01	73.28	4.51	1.03	−2.41
FME3	0.88	1.23	3.08	5.42	19.95	69.46	4.55	0.83	−2.17
SPA1	0.51	0.12	0.49	0.74	7.27	**90.89**	4.89	0.4	**−4.81**
SPA2	0.63	0.86	1.97	1.72	16.50	78.33	4.7	0.69	**−3.01**
SPA3	1.01	0.37	0.49	0.74	9.24	88.18	4.86	0.46	**−4.53**
SPA4	1.77	2.59	5.05	6.16	35.34	49.14	4.25	0.97	−1.56
SPA5	0.88	0.37	0.00	2.09	11.95	84.73	4.82	0.49	**−3.53**
SPA6	1.01	2.34	4.06	3.33	21.18	68.10	4.5	0.92	−2.22
LSA1	0.25	0.00	0.37	0.37	5.79	**93.23**	4.92	0.32	**−5.16**
LSA2	0.76	0.00	1.23	2.83	12.07	83.13	4.78	0.55	−2.87
LSA3	0.88	0.37	1.35	2.34	14.66	80.42	4.75	0.6	**−3.07**
LSA4	1.14	1.72	2.71	3.94	16.38	74.14	4.6	0.83	−2.52
CPA1	0.76	0.25	0.12	1.35	9.11	88.42	4.87	0.43	−4.2
CPA2	1.39	1.11	5.67	9.36	39.66	42.86	4.19	0.91	−1.21
CPA3	0.88	0.62	2.71	5.54	25.62	64.66	4.53	0.77	−1.92
CPA4	0.51	0.49	1.35	1.48	10.84	85.34	4.8	0.58	**−3.72**
SBI1	1.39	46.92	30.54	6.03	9.61	5.30	1.94	1.19	1.24
SBI2	0.51	73.40	16.75	2.46	4.80	2.09	1.45	0.92	2.37
SV1	0.13	1.97	0.74	0.62	8.62	87.93	4.8	0.68	**−4.28**
SV2	0.13	52.71	26.97	3.20	12.19	4.80	1.9	1.22	1.26
SV3	0.13	1.35	2.34	1.23	13.55	81.40	4.71	0.74	**−3.23**
SV4	0.38	44.58	18.84	1.85	26.72	7.64	2.34	1.46	0.52
SV5	0.13	63.42	16.50	5.30	10.84	3.82	1.75	1.19	1.44
IMS1	1.01	2.71	1.11	0.12	5.05	**90.02**	4.8	0.75	**−4.26**
IMS2	**20.48**	7.27	5.05	2.96	17.49	46.92	4.15	1.3	−1.46
IMS3	**22.38**	13.67	7.14	3.69	15.39	37.68	3.73	1.55	−0.82
IMS4	2.65	1.72	1.11	0.99	11.33	82.14	4.76	0.7	**−3.76**
IMS5	0.51	0.74	1.11	0.37	7.76	89.53	4.85	0.54	**−4.8**
IRS1	0.00	1.11	4.93	0.99	29.80	63.18	4.48	0.84	−2.07
IRS2	0.51	51.72	30.05	1.60	11.08	5.05	1.86	1.19	1.38
IRS3	0.25	0.37	0.49	0.86	8.50	89.53	4.87	0.45	**−4.8**
IRS4	3.16	23.52	18.72	7.39	18.10	29.19	3.09	1.6	−0.07
IRS5	0.13	87.93	6.53	0.99	2.46	1.97	1.24	0.76	**3.68**

Bold means possible characteristics to delete items.

**Table 2:** Goodness-of-fit index in different EFAs.

Model	Chi^2^ Base	Chi^2^	df	RMSEA	90 CI	CFI	TLI	Variance Explained
EFA 55 ITEMS 12 FACTORS	16,494.13	1101.928	891	0.017	(0.014, 0.021)	0.986	0.977	57.1%
EFA 55 ITEMS 13 FACTORS	16,494.13	1001.915	848	0.015	(0.011, 0.019)	0.990	0.982	58.3%
EFA 55 ITEMS 14 FACTORS	16,494.13	908.883	806	0.013	(0.007, 0.017)	0.993	0.987	59.7%
EFA 51 ITEMS 14 FACTORS	14,310.81	708.764	652	0.011	(0.004, 0.016)	0.996	0.991	60.6%
EFA 48 ITEMS 14 FACTORS	**12,769.16**	**562.331**	**547**	**0.006**	**(0.001, 0.012)**	**0.998**	**0.996**	**61.3%**

Bold shows the selected model.

**Table 3:** Scale scores and dimensions: description and reliability (internal consistency).

	# Of Items	Range	Mean Item	Sum Mean	Alpha Cronbach
F1	2	2–15	3.77	7.54	0.84
F2	4	4–20	4.15	16.60	0.93
F3	5	5–25	3.37	16.87	0.9
F4	6	10–30	4.60	27.58	0.94
F5	3	3–15	4.34	13.01	0.97
F6	3	3–15	3.60	10.81	0.88
F7	2	2–10	4.57	9.14	0.8
F8	3	3–15	4.27	12.81	0.92
F9	3	3–15	4.79	14.37	0.97
F10	8	8–40	4.67	37.33	0.97
F11	2	2–10	4.16	8.31	0.94
F12	2	2–10	4.75	9.50	0.9
F13	3	3–15	4.74	14.21	0.94
F14	2	2–10	4.67	9.34	0.72
TOTAL	48	125–238	4.30	207.42	0.89

**Table 4:** Final structure of PRELSA Scale with 14 dimensions.

	CWC	REW	PRE	VQD	MOV	SMT	FME	DIS	IPA	SCP	MOT	LSV	IMS	IRS	R2
CWC2	0.74														0.548
CWC4	0.661														0.437
RES3		0.726													0.527
RES4		0.707													0.5
SBI2		0.685													0.469
SV4		0.659													0.435
PRE1			0.766												0.587
PRE2			0.65												0.423
PRE3			0.795												0.632
PRE4			0.685												0.47
FME4			0.335												0.112
RES2				0.687											0.472
VQD1				0.55											0.303
VQD2				−0.686											0.47
VQD3				0.603											0.364
VQD4				0.729											0.532
FME3				0.779											0.607
SME1					0.894										0.8
SME3					−0.843										0.71
SME5					0.743										0.552
SMT1						0.824									0.68
SMT2						0.547									0.299
SMT3						−0.557									0.311
FME1							0.715								0.511
FME2							0.718								0.516
SME2								0.867							0.751
SME4								0.648							0.419
SV5								0.914							0.835
SPA1									0.913						0.834
SPA2									0.798						0.636
SPA3									0.875						0.766
SPA5										0.856					0.732
SPA6										0.649					0.422
LSA2										0.697					0.486
LSA3										0.749					0.56
LSA4										0.54					0.291
CPA1										0.598					0.358
CPA3										0.698					0.487
CPA4										0.774					0.598
SPA4											0.762				0.58
CPA2											0.848				0.72
SV1												0.782			0.612
SV3												0.738			0.545
IMS1													0.687		0.472
IMS4													0.769		0.591
IMS5													0.91		0.829
IRS1														0.501	0.251
IRS3														0.694	0.481
	Correlations between Factors														
	CWC	REW	PRE	VQD	MOV	SMT	FME	DIS	IPA	SCP	MOT	LSV	IMS	IRS	
CWC	1														
REW	0.052	1													
PRE	0.101	0.563 *	1												
VQD	0.083	−0.528 *	−0.342 *	1											
MOV	−0.088	−0.081	−0.388 *	−0.002	1										
SMT	−0.242	−0.186 *	−0.549 *	0.166 *	0.648 *	1									
FME	0.095	−0.241 *	−0.104	0.493 *	−0.227 *	−0.079	1								
DIS	−0.025	0.664 *	0.399 *	−0.492 *	0.027	−0.105	−0.294 *	1							
IPA	0.05	−0.3 *	−0.228 *	0.546 *	0.037	0.059	0.345 *	−0.207 *	1						
SCP	0.09	−0.273 *	−0.295 *	0.61 *	−0.004	0.147 *	0.41 *	−0.235 *	0.828 *	1					
MOT	0.121 *	−0.008	0.057	0.339 *	−0.029	−0.06	0.237 *	−0.049	0.577 *	0.675 *	1				
LSV	0.112 *	−0.385 *	−0.255 *	0.651 *	−0.046	0.074	0.417 *	−0.399 *	0.513 *	0.552 *	0.224 *	1			
IMS	0.046	−0.297 *	−0.166 *	0.495 *	−0.11 *	0.01	0.415 *	−0.299 *	0.522 *	0.643 *	0.241 *	0.581 *	1		
IRS	0.013	−0.104	0.098	0.319 *	−0.299 *	−0.229 *	0.416 *	−0.051	0.351 *	0.405 *	0.326 *	0.359 *	0.522 *	1	

* Significant at 0.01% level. CWC: Child’s Weight Concern, REW: Use of Rewards, PRE: Pressure to Eat, VQD: Variety and Quality of Diet, MOV: Movement during Meal, SMT: Structured Mealtime, FME: Family Meal Environment, DIS: Distractions during Mealtime, IPA: Importance of Physical Activity, MOT: Motivation to Physical Activity, SCP: Support and Co-Participation in Physical Activity, LSV: Limits Screen Viewing, IMS: Impact and Modification of Sleep, IRS: Interactions regarding Sleep.

**Table 5:** Goodness-of-fit index in multigroup CFA models.

Variable	Model	CFI	RMSEA	Cfi Diff	RMSEA Diff
Education	Configural Model	0.932	0.031		
	Metric Model	0.932	0.031	0	0
	Scalar Model	0.932	0.030	0	0.001
Income	Configural Model	0.935	0.031		
	Metric Model	0.937	0.030	0.002	0.001
	Scalar Model	0.937	0.030	0	0
Marital Status	Configural Model	0.927	0.032		
	Metric Model	0.929	0.032	0	0
	Scalar Model	0.929	0.031	0	0.001

## Data Availability

Data are unavailable due to privacy or ethical restrictions.

## Supplementary Materials

The following supporting information can be downloaded at: https://www.mdpi.com/article/10.3390/nu16081135/s1, Table S1: Demographic Characteristics in Field Test. Table S2: Results of the chosen EFA Model (48 Items—14 Dimensions).
