# Supplementary material for: The Dimensionality, Consistency, and Structural Validity of an Instrument Used to Measure Obesogenic Attitudes in Parents from Southern Spain (The PRELSA Scale)
Source: Nutrients. 2024 Apr 11;16(8):1135. doi: 10.3390/nu16081135 (PMC11053490; doi:10.3390/nu16081135)
Supplement: Supplementary file 1 [file nutrients-16-01135-s001.zip › nutrients-2950461-supplementary.pdf]

The Dimensionality, Consistency, and Structural Validity of an Instrument Used to Measure Obesogenic Attitudes in Parents from Southern Spain (The PRELSA Scale)

Supplementary Material:

Table S1: Demographic Characteristics in Field Test

VARIABLE	CATEGORIES	N	%
**SEX**	Women	738	93.3
	Men	53	6.7
**MARITAL STATUS**	Married	536	67.8
	Divorced	18	2.2
	Domestic Partner	92	11.6
	Single	7	0.8
**RELATION WITH THE CHILD**	Parents	784	99.1
	Family and Caregiver	4	0.5
	No Family and Caregiver	3	0.4
**EDUCATION LEVEL**	Primary	34	4.3
	Secondary	59	7.4
	Bachelor	48	6.1
	Professional Training	165	20.8
	University Degree	334	42.2
	Master	128	16.2
	PhD	23	2.9
**JOB SITUATION**	Full Time Job	403	50.9
	Partial Time Job	191	24.1
	Unemployed	186	23.5
	Other situation	11	1.4
**INCOME**	<1000€	58	7.3
	1000€-2000€	301	38.1
	2000€-4000€	346	43.7
	More than 4000 €	80	10.1
	Mean	SD	IC
**AGE**	37.95	5.17	(27.8, 48.1)
**FAMILY MEMBERS**	3.68	0.81	(2.1, 5.3)
**CHILDREN AGE**	3.79	1.41	(1.0, 6.5)
**IMC PARENTS**	25.53	5.45	(14.8, 36.2)
**Z-IMC/AGE CHILDREN**	0.18	1.40	(-2.6, 2.9)

Table S2: Results of the chosen EFA Model (48 Items - 14 Dimensions)

	F1	F2	F3	F4	F5	F6	F7	F8	F9	F10	F11	F12	F13	F14	COMM
**CWP2**	*0.872*	-0.077	0.069	0.02	0.112	-0.093	0.028	-0.137	0.069	-0.062	0.138	0.007	-0.074	0.007	0.753
**CWP4**	*0.636*	-0.037	0.029	-0.09	-0.005	0.155	-0.007	0.085	0.021	0.018	-0.035	0.124	-0.032	0.023	0.391
**RES2**	-0.083	-0.125	0.238	*0.386*	-0.039	0.006	-0.085	0.042	-0.104	0.001	0.21	0.254	-0.196	0.026	0.54
**RES3**	-0.049	*0.634*	0.059	-0.034	-0.16	-0.1	0.044	0.049	0.142	-0.058	-0.035	0.047	0.009	-0.043	0.57

**RES4**	-0.025	*0.93*	-0.022	-0.049	0.032	-0.006	-0.022	-0.067	-0.066	-0.087	0.15	-0.041	-0.091	-0.017	0.832
**PRE1**	0.046	0.061	*0.629*	-0.105	-0.026	0.012	0.252	0	-0.042	0.028	-0.112	-0.087	-0.084	-0.04	0.586
**PRE2**	0.014	0.127	*0.573*	-0.039	-0.113	-0.048	0.096	-0.095	0.002	0.074	-0.034	0.191	0.038	-0.113	0.53
**PRE3**	-0.008	-0.025	*0.731*	-0.121	0.059	-0.065	-0.061	-0.024	-0.031	0.1	-0.111	-0.047	0.001	0.016	0.658
**PRE4**	0.038	-0.008	*0.745*	0.088	-0.041	-0.04	-0.029	-0.16	0.099	-0.088	0.094	-0.007	0.073	-0.014	0.6
**VQD1**	0	-0.202	0.138	*0.566*	-0.009	-0.021	-0.247	0.059	-0.116	0.051	-0.135	0.008	-0.042	0.156	0.524
**VQD2**	0.029	0.145	0.023	*-0.38*	-0.062	0.129	0.012	0.011	0.091	0.156	-0.291	-0.059	0.08	-0.091	0.458
**VQD3**	-0.031	-0.038	-0.039	*0.962*	-0.093	0.011	0.044	-0.092	0.076	0.042	-0.135	-0.084	0.025	-0.11	0.641
**VQD4**	-0.026	0.108	0.098	*0.547*	-0.068	0.078	0.013	0.102	-0.117	-0.196	0.26	0.026	-0.106	-0.001	0.583
**SME1**	0.045	0.019	-0.019	-0.027	*0.959*	0.042	0.091	0.078	0.017	0.047	-0.171	0.071	0.131	-0.081	0.886
**SME2**	0.049	-0.041	0.122	-0.009	-0.087	0.016	-0.099	0.164	*0.909*	-0.071	0.094	0.019	-0.17	0.075	0.794
**SME3**	0.04	0.114	0.034	0.159	*-0.771*	-0.01	0.106	-0.073	0.058	0.05	-0.065	-0.032	-0.075	0.141	0.723
**SME4**	-0.02	0.096	0.067	0.146	*0.312*	-0.073	-0.086	-0.034	*0.739*	-0.089	0.212	-0.007	-0.075	0.037	0.607
**SME5**	0.081	0.055	0.042	0.021	*0.722*	0.161	-0.113	0.045	0.013	0.042	-0.101	0.041	-0.016	0.066	0.64
**SMT1**	0.023	-0.063	-0.111	0.084	0.108	*0.699*	0.076	-0.117	0.015	0.028	0.16	-0.094	-0.149	0.122	0.685
**SMT2**	-0.05	0.049	0.021	-0.118	0.044	*0.69*	0.076	-0.071	-0.063	-0.043	-0.086	0.153	0.059	-0.026	0.503
**SMT3**	0.061	0.142	0.131	0.054	-0.018	*-0.334*	0.054	0.08	0.126	-0.043	-0.178	0.1	0.281	0.155	0.384
**SMT4**	-0.011	0.017	*0.358*	0.026	0.26	0.189	0.044	0.13	0.002	-0.002	-0.296	-0.264	0.04	0.091	0.336
**FME1**	0.024	0.075	0.041	0.065	-0.012	0.09	*0.799*	0.047	-0.095	-0.009	0.109	0.018	-0.011	-0.008	0.717
**FME2**	0.11	-0.066	-0.045	0.064	-0.02	0.002	*0.509*	0.252	-0.071	-0.088	-0.156	0.089	-0.033	0.25	0.479
**FME3**	0.048	-0.068	-0.131	*0.726*	0.001	-0.045	0.18	-0.121	0.104	0.112	0.036	0.054	0.11	-0.085	0.665
**SPA1**	0.033	-0.084	-0.065	-0.119	0.002	-0.067	0.077	*1.067*	0.094	-0.105	0.147	-0.079	-0.113	0.056	0.932
**SPA2**	-0.094	-0.045	-0.077	-0.044	0.184	-0.084	0.073	*0.999*	0.055	0.02	-0.102	0.023	-0.066	-0.001	0.776
**SPA3**	0.07	-0.143	0.181	0.052	-0.126	0.155	-0.127	*0.432*	-0.021	0.085	0.296	-0.035	0.154	*-0.341*	0.831
**SPA4**	0.062	-0.061	0.014	0.107	-0.037	0.061	-0.126	0.05	-0.058	*0.807*	-0.111	0.015	0.023	0.069	0.67
**SPA5**	0.058	0.033	0	0.09	-0.058	0.049	0.004	0.204	-0.061	0.087	*0.384*	0.077	0.198	-0.228	0.722
**SPA5**	0.103	0.052	-0.145	-0.095	-0.103	-0.034	-0.041	0.038	-0.08	0.064	*0.716*	-0.094	-0.04	-0.012	0.516
**LSA2**	0.059	0.084	-0.019	0.017	-0.073	0.131	0.006	0.064	-0.04	0.106	*0.45*	0.043	0.113	-0.071	0.496
**LSA3**	0.021	0.08	0	0.017	0.029	-0.027	-0.113	0.23	-0.018	0.032	*0.542*	0.019	0.039	-0.032	0.581
**LSA4**	-0.041	0.085	-0.005	0.055	-0.032	0.028	0.151	-0.108	0.061	-0.024	*0.57*	-0.028	0.154	-0.103	0.381
**CPA1**	-0.026	-0.229	0.007	-0.122	-0.071	0.13	0.085	-0.129	0.19	-0.132	*0.717*	0.031	0.169	-0.025	0.568
**CPA2**	-0.09	-0.078	0.037	-0.002	0.136	-0.112	0.063	-0.15	-0.039	*0.813*	0.253	0.058	-0.057	0.104	0.774
**CPA3**	0.006	-0.021	0.042	-0.081	0.086	-0.127	0.141	0.086	0.083	0.13	*0.728*	0.021	-0.074	-0.067	0.62
**CPA4**	-0.038	0.162	0.022	0.053	-0.042	0.058	-0.027	-0.016	-0.074	-0.011	*0.807*	-0.046	-0.022	0.142	0.709
**SBI2**	0.002	*0.5*	0.018	0.028	0.058	0.146	0.052	-0.064	0.128	0.136	-0.269	-0.024	0.058	-0.088	0.497
**SV1**	-0.04	0.114	-0.031	0.136	0.068	-0.092	-0.092	0.16	-0.05	-0.133	-0.098	*0.526*	0.269	-0.058	0.545
**SV3**	0.043	0.051	-0.013	-0.081	0.069	0.039	0.031	-0.091	-0.069	0.058	-0.038	*1.025*	-0.088	0.083	0.87
**SV4**	0.023	*0.359*	-0.021	0.025	-0.13	0.151	-0.092	-0.03	*0.353*	0.125	-0.192	-0.032	0.167	-0.031	0.448
**SV5**	-0.004	0.031	-0.054	0.077	-0.05	-0.033	-0.054	0.064	*0.95*	0.024	-0.076	-0.072	0.066	0.095	0.841
**IMS1**	-0.072	-0.03	0.046	0.13	0.085	0.025	-0.075	-0.08	0.111	0.014	0.078	0.103	*0.564*	0.119	0.462
**IMS4**	-0.026	-0.056	0.064	-0.039	0.058	-0.054	0.02	-0.086	-0.064	0.041	0.02	-0.062	*0.929*	0.003	0.763
**IMS5**	0.004	-0.009	-0.003	0.041	0.122	-0.053	-0.083	-0.055	-0.077	-0.125	0.226	-0.041	*0.736*	0.164	0.771
**IRS1**	-0.062	-0.051	0.052	0.028	0.013	-0.087	-0.042	0.078	0.108	0.081	0.135	0.015	-0.03	*0.382*	0.227
**IRS3**	0.029	-0.078	-0.016	-0.096	-0.092	0.07	0.033	0.061	0.085	0.098	-0.009	0.061	0.108	*0.713*	0.588
	CORRELATIONS BETWEEN FACTORS														
**F1**	1														
**F2**	0.134	1													
**F3**	0.085	0.408	1												
**F4**	0.117	-0.227	-0.127	1											
**F5**	-0.172	0.009	-0.351	0.051	1										
**F6**	-0.24	-0.045	-0.322	0.232	0.457	1									
**F7**	-0.012	-0.082	-0.039	0.121	-0.166	-0.116	1								
**F8**	0.093	-0.1	-0.003	0.544	-0.1	0.146	0.079	1							
**F9**	-0.087	0.393	0.257	-0.499	0.006	-0.04	0.04	-0.315	1						
**F10**	0.146	0.197	0.179	0.174	-0.078	-0.032	0.135	0.483	0.055	1					
**F11**	0.033	-0.176	-0.168	0.54	0.009	0.249	0.146	0.704	-0.316	0.468	1				
**F12**	0.031	-0.291	-0.081	0.487	-0.157	0.12	0.168	0.492	-0.335	0.131	0.52	1			
**F13**	0.097	-0.154	-0.002	0.376	-0.242	0.144	0.253	0.552	-0.243	0.167	0.565	0.454	1		
**F14**	0.04	0.055	0.141	0.244	-0.17	-0.076	0.063	0.16	-0.219	-0.06	0.159	0.176	0.276	1	

Preschool Eating, Lifestyle, and Sleeping Attitudes Scale (PRELSA Scale)
English Version (60 items and 13 dimensions)

Part 1: Feeding Practices and Attitudes

Child’s Weight Concern and Perception (CWC)

- **CWC1**: Do you think your child currently presents...? *Answers: 1 = very low weight, 2 = low weight, 3 = normal weight, 4 = overweight, 5 = obesity*

Answers: 1 = not worried at all, 2 = a little worried, 3 = worried, 4 = quite worried, 5 = very worried

- **CWC2:** Are you worried that your child eats too much?
- **CWC3:** Are you worried that your child is overweight during childhood?
- **CWC4:** Are you concerned that potential excess weight in your child during this preschool stage could be a health problem in the short/medium term?

Restrictions

Answers: 1 = disagree, 2 = slightly disagree, 3 = indifferent/don't care, 4 = slightly agree, 5 = agree, 9 = NS/NC

- **RES1:** I consider it important to control my child's intake of sweets (cookies, cakes, candies).
- **RES2:** I consider it important to control my child's intake of high-calorie foods (like snacks, pizzas, and/or hamburgers).
- **RES3:** I consider it appropriate to use some type of food as a reward when behaving well.
- **RES4:** I consider it appropriate to use food to calm my child down.

Pressure to eat

Answers: 1 = disagree, 2 = slightly disagree, 3 = indifferent/don't care, 4 = slightly agree, 5 = agree, 9 = NS/NC

- **PRE1:** I believe my child should always finish all the food on the plate.
- **PRE2:** I try to make sure my child eats what I consider necessary.
- **PRE3:** I try to make my child eat regardless of whether they say they are not hungry.
- **PRE4:** I try to control my child's food intake so that they don't eat less than they should.

Variety and quality of the diet

Answers: 1 = disagree, 2 = slightly disagree, 3 = indifferent/don't care, 4 = slightly agree, 5 = agree, 9 = NS/NC

- **VQD1:** I consider it appropriate to offer water as the only drink for my child at main meals.
- **VQD2:** I consider it appropriate to offer my child pre-cooked and/or industrially processed foods in their regular diet.
- **VQD3:** I consider it necessary to always offer salads and/or vegetables in my child's meals.
- **VQD4:** I consider it necessary for my child to eat fruits every day.
- **VQD5:** I consider it necessary for my child to eat enough, even if the diet is sometimes not varied.

Structured Meal Environment

Answers: 1 = disagree, 2 = slightly disagree, 3 = indifferent/don't care, 4 = slightly agree, 5 = agree, 9 = NS/NC

- **SME1:** I consider it appropriate for my child to get up or move around during meals.
- **SME2:** I consider it appropriate for my child to eat entertained with screens (TV, computer, mobiles, and/or tablets).
- **SME3:** I consider it appropriate for my child to remain seated throughout mealtime.
- **SME4:** I consider it appropriate for my child to eat entertained with some game.
- **SME5:** I consider it appropriate for my child to eat if they are not seated at the table.

Structured Mealtime

Answers: 1 = disagree, 2 = slightly disagree, 3 = indifferent/don't care, 4 = slightly agree, 5 = agree, 9 = NS/NC

- **SMT1:** I consider it appropriate for my child to decide when they want to eat.
- **SMT2:** I consider it appropriate for my child to sometimes eat between meals.
- **SMT3**: I am the one who should organize my child's meal schedules.
- **SMT4**: I consider it appropriate for my child to eat quickly.

Family Meal Environment

Answers: 1 = disagree, 2 = slightly disagree, 3 = indifferent/don't care, 4 = slightly agree, 5 = agree, 9 = NS/NC

- **FME1:** I consider it necessary for my child to eat main meals with the rest of the family.
- **FME2**: I consider it necessary for my child to eat the same food as the rest of the family.
- **FME3**: I consider it necessary for family meals to always have dishes with salads and/or vegetables on the table.

Total of 29 items

Part 2: Physical Activity, Sedentarism, and Screen Time Attitudes

Stimulate Physical Activity

Answers: 1 = disagree, 2 = slightly disagree, 3 = indifferent/don't care, 4 = slightly agree, 5 = agree, 9 = NS/NC

- **SPA1:** I consider my child's physical activity as important for their development as their nutrition.
- **SPA2:** I consider my child's physical activity as important for their development as their studies.
- **SPA3:** I try to teach my child that being active is good for their health.
- **SPA4**: I consider it appropriate to motivate my child to engage in physical activities over other types of activities.
- **SPA5:** I consider it important to introduce my child to various options for physical activity and active play.
- **SPA6**: I believe my child can learn healthy habits of physical activity through my example.

Logistic Support for Activity (environment, materials...)

Answers: 1 = disagree, 2 = slightly disagree, 3 = indifferent/don't care, 4 = slightly agree, 5 = agree, 9 = NS/NC

- **LSA1**: I consider it necessary to take my child to places that have areas or parks for children.
- **LSA2**: I consider it necessary to provide my child with toys and/or games that encourage physical activity.
- **LSA3**: I consider it necessary to organize my child's routines so that there is frequently space for physical activity.
- **LSA4**: One of my priorities when thinking about where to live is that my child has spaces to play indoors or outdoors.

Co-Participation in Activity

Answers: 1 = disagree, 2 = slightly disagree, 3 = indifferent/don't care, 4 = slightly agree, 5 = agree, 9 = NS/NC

- **CPA1**: I consider it important to ask my child what they like when it comes to playing.
- **CPA2**: I consider it appropriate to play active games and/or sports with my child over other activities.
- **CPA3**: I believe it's necessary to have time for physical activity with my child.
- **CPA4**: I believe family habits of physical activity can influence my child's healthy lifestyle throughout their life.

<u>Sedentary Behavior Issues</u>

Answers: 1 = disagree, 2 = slightly disagree, 3 = indifferent/don't care, 4 = slightly agree, 5 = agree, 9 = NS/NC

- **SBI1**: I consider it appropriate to habitually keep my child distracted through activities that don't require physical effort.
- **SBI2**: I consider it appropriate to offer rewards to my child to keep them still.

<u>Screen Viewing (mobiles, tablets, computers, television)</u>

Answers: 1 = disagree, 2 = slightly disagree, 3 = indifferent/don't care, 4 = slightly agree, 5 = agree, 9 = NS/NC

- **SV1**: I consider it appropriate to limit my child's screen viewing (TV, computer, mobiles, and/or tablets).
- **SV2**: I consider it appropriate for screen viewing to be part of my child's regular routine.
- **SV3**: I consider it appropriate to limit my screen time when I'm with my child/children.
- **SV4**: I consider it appropriate to occasionally use screens to calm and/or reward my child.
- **SV5**: I consider it appropriate to use screens during meals.

Total of 21 items

<u>Part 3: Sleeping Attitudes</u>

<u>Impact and Modification of Sleep</u>

Answers: 1 = disagree, 2 = slightly disagree, 3 = indifferent/don't care, 4 = slightly agree, 5 = agree, 9 = NS/NC

- **IMS1:** I believe sleep routines affect other areas of my child's mental and physical development.
- **IMS2:** I believe sleep problems can be improved by changing children's habits.
- **IMS3:** I believe sleep problems in my child can cause obesity problems in the future.
- **IMS4:** I believe my child's lack of sleep can encourage them to eat more during the day.
- **IMS5:** I believe family routines can influence proper sleep patterns for children.

<u>Interactions Regarding Sleep</u>

Answers: 1 = disagree, 2 = slightly disagree, 3 = indifferent/don't care, 4 = slightly agree, 5 = agree, 9 = NS/NC

- **IRS1:** I consider it appropriate for my child to have the same sleep habits every day of the week.
- **IRS2:** I consider it appropriate for my child to make decisions about their sleep routines and schedules.
- **IRS3:** I consider it appropriate for my child to sleep most nights in the same place.
- **IRS4:** I consider it appropriate for my child to frequently come to my bed if they wake up at night.
- **IRS5:** I consider it appropriate for my child to use screens (TV, computer, mobiles, and/or tablets) to fall asleep.

Total of 10 items

Preschool Eating, Lifestyle, and Sleeping Attitudes Scale (PRELSA Scale)
SECOND VERSION (48 ITEMS-14 DIMENSIONS)

Child's Weight Concern (CWC)

Answers: 1 = not worried at all, 2 = a little worried, 3 = worried, 4 = quite worried, 5 = very worried

- **CWC2:** Are you worried that your child eats too much?
- **CWC4:** Are you concerned that potential excess weight in your child during this preschool stage could be a health problem in the short/medium term?

Use of Rewards (REW)

Answers: 1 = disagree, 2 = slightly disagree, 3 = indifferent/don't care, 4 = slightly agree, 5 = agree, 9 = NS/NC

- **RES3:** I consider it appropriate to use some type of food as a reward when behaving well.
- **RES4:** I consider it appropriate to use food to calm my child down.
- **SBI2**: I consider it appropriate to offer rewards to my child to keep them still.

- **SV4**: I consider it appropriate to occasionally use screens to calm and/or reward my child.

Pressure to eat (PRE)

Answers: 1 = disagree, 2 = slightly disagree, 3 = indifferent/don't care, 4 = slightly agree, 5 = agree, 9 = NS/NC

- **PRE1:** I believe my child should always finish all the food on the plate.
- **PRE2:** I try to make sure my child eats what I consider necessary.
- **PRE3:** I try to make my child eat regardless of whether they say they are not hungry.
- **PRE4:** I try to control my child's food intake so that they don't eat less than they should.
- **VQD5:** I consider it necessary for my child to eat enough, even if the diet is sometimes not varied.

Variety and Quality of the Diet (VQD)

Answers: 1 = disagree, 2 = slightly disagree, 3 = indifferent/don't care, 4 = slightly agree, 5 = agree, 9 = NS/NC

- **RES2:** I consider it important to control my child's intake of high-calorie foods (like snacks, pizzas, and/or hamburgers).
- **VQD1:** I consider it appropriate to offer water as the only drink for my child at main meals.
- **VQD2:** I consider it appropriate to offer my child pre-cooked and/or industrially processed foods in their regular diet.
- **VQD3:** I consider it necessary to always offer salads and/or vegetables in my child's meals.
- **VQD4:** I consider it necessary for my child to eat fruits every day.
- **FME3:** I consider it necessary for family meals to always have dishes with salads and/or vegetables on the table.

Movement during Mealtime (MOV)

Answers: 1 = disagree, 2 = slightly disagree, 3 = indifferent/don't care, 4 = slightly agree, 5 = agree, 9 = NS/NC

- **SME1:** I consider it appropriate for my child to get up or move around during meals.
- **SME3:** I consider it appropriate for my child to remain seated throughout mealtime.
- **SME5:** I consider it appropriate for my child to eat if they are not seated at the table.

Structured Mealtime (SMT)

Answers: 1 = disagree, 2 = slightly disagree, 3 = indifferent/don't care, 4 = slightly agree, 5 = agree, 9 = NS/NC

- **SMT1:** I consider it appropriate for my child to be able to decide when he/she wants to eat.
- **SMT2:** I consider it appropriate for my child to sometimes eat between routine meals.
- **SMT3**: I am the one who should organize my child's meal schedules.

Family Meal Environment (FME)

Answers: 1 = disagree, 2 = slightly disagree, 3 = indifferent/don't care, 4 = slightly agree, 5 = agree, 9 = NS/NC

- **FME1:** I consider it necessary for my child to eat main meals with the rest of the family.
- **FME2**: I consider it necessary for my child to eat the same food as the rest of the family.

Distractions during Mealtime (DIS)

Answers: 1 = disagree, 2 = slightly disagree, 3 = indifferent/don't care, 4 = slightly agree, 5 = agree, 9 = NS/NC

- **SME2:** I consider it appropriate for my child to eat entertained with screens (TV, computer, mobiles, and/or tablets).
- **SME4:** I consider it appropriate for my child to eat entertained with some game.
- **SV5**: I consider it appropriate to use screens during meals.

Importance of Physical Activity (IPA)

Answers: 1 = disagree, 2 = slightly disagree, 3 = indifferent/don't care, 4 = slightly agree, 5 = agree, 9 = NS/NC

- **SPA1:** I consider my child's physical activity as important for their development as their nutrition.
- **SPA2:** I consider my child's physical activity as important for their development as their studies.
- **SPA3:** I try to teach my child that being active is good for their health.

Support and Co-Participation in Physical Activity (SCP)

Answers: 1 = disagree, 2 = slightly disagree, 3 = indifferent/don't care, 4 = slightly agree, 5 = agree, 9 = NS/NC

- **SPA5:** I consider it important to introduce my child to various options for physical activity and active play.
- **SPA6:** I believe my child can learn healthy habits of physical activity through my example.
- **LSA2**: I consider it necessary to provide my child with toys and/or games that encourage physical activity.

- **LSA3**: I consider it necessary to organize my child's routines so that there is frequently space for physical activity.
- **LSA4**: One of my priorities when thinking about where to live is that my child has spaces to play indoors or outdoors.
- **CPA1**: I consider it important to ask my child what they like when it comes to playing.
- **CPA3**: I believe it's necessary to have time for physical activity with my child.
- **CPA4**: I believe family habits of physical activity can influence my child's healthy lifestyle throughout their life.

Motivation to Physical Activity (MOT)

Answers: 1 = disagree, 2 = slightly disagree, 3 = indifferent/don't care, 4 = slightly agree, 5 = agree, 9 = NS/NC

- **SPA4**: I consider it appropriate to motivate my child to engage in physical activities over other types of activities.
- **CPA2**: I consider it appropriate to play active games and/or sports with my child over other activities.

Limits Screen Viewing (LSV)

Answers: 1 = disagree, 2 = slightly disagree, 3 = indifferent/don't care, 4 = slightly agree, 5 = agree, 9 = NS/NC

- **SV1**: I consider it appropriate to limit my child's screen viewing (TV, computer, mobiles, and/or tablets).
- **SV3**: I consider it appropriate to limit my screen time when I'm with my child/children.

Impact and Modification of Sleep (IMS)

Answers: 1 = disagree, 2 = slightly disagree, 3 = indifferent/don't care, 4 = slightly agree, 5 = agree, 9 = NS/NC

- **IMS1:** I believe sleep routines affect other areas of my child's mental and physical development.
- **IMS4:** I believe my child's lack of sleep can encourage them to eat more during the day.
- **IMS5:** I believe family routines can influence proper sleep patterns for children.

Importance of Routines regarding Sleep (IRS)

Answers: 1 = disagree, 2 = slightly disagree, 3 = indifferent/don't care, 4 = slightly agree, 5 = agree, 9 = NS/NC

- **IS1:** I consider it appropriate for my child to have the same sleep habits every day of the week.
- **IS3:** I consider it appropriate for my child to sleep most nights in the same place.
